# Dephosphorylation of Y685-VE-Cadherin Involved in Pulmonary Microvascular Endothelial Barrier Injury Induced by Angiotensin II

**DOI:** 10.1155/2016/8696481

**Published:** 2016-12-29

**Authors:** Zhiyong Wu, Zhiwei Wang, Feifeng Dai, Huagang Liu, Wei Ren, Jinxing Chang, Bowen Li

**Affiliations:** Department of Cardiovascular Surgery, Renmin Hospital of Wuhan University, Wuhan 430060, China

## Abstract

Angiotensin II (AngII) caused pulmonary microvascular endothelial barrier injury, which induced acute aortic dissection (AAD) combined with acute lung injury (ALI). However, the exact mechanism is unclear. We investigated the role of dephosphorylation of Y685-VE-cadherin in the AngII induced pulmonary microvascular endothelial barrier injury. Mice or pulmonary microvascular endothelial cells (PMVECs) were divided into control group, AngII group, AngII+PP2 (Src kinase inhibitor) group, and PP2 group. PP2 was used to inhibit the phosphorylation of Y685-VE-cadherin. Pathological changes, infiltration of macrophages and neutrophils, and pulmonary microvascular permeability were used to determine the pulmonary microvascular endothelial barrier function. Flow cytometry was used to determine the apoptosis of PMVECs, and immunofluorescence was used to determine the skeletal arrangement. Transendothelial resistance was used to detect the permeability of endothelial barrier. Phosphorylation of Y685-VE-cadherin was significantly reduced after AngII stimulation (*P* < 0.05), together with skeletal rearrangement, and elevation of endothelial permeability which finally induced endothelial barrier injury. After PP2 interference, the phosphorylation of Y685-VE-cadherin was further reduced and the endothelial permeability was further elevated. These data indicated that AngII could induce pulmonary injury by triggering endothelial barrier injury, and such process may be related to the dephosphorylation of Y685-VE-cadherin and the endothelial skeletal rearrangement.

## 1. Introduction

Aortic dissection (AD), with a high mortality worldwide, causes great threats to the public health [1]. Our previous study showed that a large number of AD patients may combine with acute lung injury (ALI), which severely affected the prognosis. Unfortunately, the exact mechanism of AAD complicated with ALI is still not well defined [2, 3]. Our previous study indicated that pulmonary microvascular endothelial barrier injury may present in patients with AAD, together with massive migration of macrophages and secretion of MMP-9, which finally induced ALI after interrupting the air-blood barrier [4]. These indicated that pulmonary microvascular endothelial barrier injury might play a crucial role in the pathogenesis of AAD complicated with ALI.

The function of vascular endothelial barrier is highly depending on the cellular adherens between the endothelial cells [5]. As an endothelial specific adhesion protein located at adherens junctions, VE-cadherin played an important role in the maintenance of vascular integrity and endothelial barrier function [6]. Under specific microenvironments, cell-cell junction interruption may be induced after phosphorylation in the tyrosine residues of VE-cadherin [7, 8]. At the same time, skeletal rearrangement of the vascular endothelial cells could induce shrinkage of endothelial cells and elevation of endothelial interspace, which further hampered the function of vascular endothelial barrier [9]. Currently, under normal conditions, the endothelial barrier function is highly depending on the connection of VE-cadherin complex in the vascular endothelial cells [10]. To the best of our knowledge, phosphorylation and dephosphorylation of intercellular tyrosine residues in the VE-cadherin complex are responsible for the modulation of VE-cadherin function [11]. Wessel et al. showed that Tyr685 and Tyr731 of VE-cadherin distinctly and selectively regulate the induction of vascular permeability or leukocyte extravasation [12]. Meanwhile, several factors have been reported to induce phosphorylation of tyrosine of the VE-cadherin including VEGF, TNF-*α*, and HIF-1*α*, which may subsequently result in elevation of vascular permeability [13–15]. Our previous study revealed that the serum Angiotensin (AngII) was remarkably elevated in AAD patients, together with elevation of pulmonary vascular permeability. Also, AngII plays an important role in the process of endothelial dysfunction in acute lung injury (ALI) [16]. In this study, we investigated the effects of AngII on the phosphorylation of Y685-VE-cadherin, as well as its roles in the pulmonary vascular endothelial barrier injury.

## 2. Materials and Methods

### 2.1. Patients and Sample

A total of 58 newly diagnosed AAD patients admitted to the intensive care unit (ICU) of our hospital between September 2014 and July 2015 were included. Among the 58 AAD patients, 21 showed concurrent hypoxemia before the surgery. Twelve matched healthy individuals were enrolled as normal control (Table 1). The diagnosis of AAD was depending on the computed tomography (CT) scan and ultrasonic examination. ALI was defined as PaO_2_/FiO_2_ ≤ 300 mmHg in the first 24 hours after diagnosis according to the diagnostic criteria by American-European Consensus Conference [17]. The exclusion criteria were as follows: (i) those admitted to our hospital 7 days or more after the onset of disease and (ii) those with cancer, chest trauma, and pulmonary infection within one month before inclusion. PaO_2_/FiO_2_ was determined within 1 h after sample collection. Written informed consent was obtained from each patient. This study was approved by the Ethical Committee of Renmin Hospital of Wuhan University.

### 2.2. Inducing ALI in Mice through Pumping of AngII

ALI mice model was established though minipumping of AngII. Male C57BL/6J mice (8 weeks old) were purchased from HFK Bioscience Co., Ltd. (Beijing, China). The mice were fed on a normal diet, combined with osmotic minipumps (Alzet, Cupertino, CA) filled with 1 *μ*g/kg per minute AngII (Sigma-Aldrich) for 1 week. All animal handling was performed in accordance with the Wuhan Directive for Animal Research and the current Guidelines for the Care and Use of Laboratory Animals published by the National Institutes of Health. Phenobarbital (40 mg/Kg, 2%) was used for the anesthesia. Extensive measures were taken to lower the suffering of the animals. The animal study was approved by the Ethical Committee of the Renmin Hospital of Wuhan University.

The animals were randomly divided into four groups, including (i) control group, (ii) AngII group, (iii) AngII+PP2 (Src kinase inhibitor), subject to AngII pumping followed by 20 *μ*g PP2 dissolved in DMSO via intraperitoneal injection, and (iv) PP2 group subject to intraperitoneal injection of 20 *μ*g PP2 dissolved in DMSO. The PP2 was injected every two days for 3 times [18].

### 2.3. Cell Culture

The rat PMVECs were purchased from BeNa Culture Collection Co., Ltd. (category No. BNCC338210; Beijing, China). Cells were cultured in RPMI 1640 medium (Invitrogen, Carlsbad, CA, USA) supplemented with 10% fetal bovine serum (FBS), 1% penicillin/streptomycin, 0.5% fungizone (Invitrogen, Carlsbad, CA, USA), and 1% endothelial cell growth supplement (ScienCell, Carlsbad, CA, USA), followed by culturing at 37°C in a humidified atmosphere of 5% CO_2_ in air. The growth of the cells was arrested by replacing 10% FBS RPMI 1640 with FBS-free RPMI 1640 for 24 h. The cells were divided into four groups, including control group; AngII group, stimulated by AngII (1 *μ*M Sigma-Aldrich, St. Louis, USA); AngII+PP2 group, stimulated by AngII (1 *μ*M Sigma-Aldrich, St. Louis, USA) and PP2 (0.1 *μ*M, Selleck Chemicals LLC, USA) as previously described [19]; and PP2 group, stimulated by PP2 (0.1 *μ*M, Selleck Chemicals LLC, USA).

### 2.4. ELISA

Serum AngII was measured using commercial ELISA kits (category number EK0459, Biofavor Biotech Service Co., Ltd., Wuhan, China) according to the manufacturer's instructions. All tests were carried out at least in triplicate.

### 2.5. Histopathological Examination

Upon collection of lung tissues, the samples were fixed and embedded. Afterwards, H&E staining and immunohistostaining were performed to determine the expression of CD68 (BioLegend, 333801) and MPO (Proteintech, 66177-1-Ig) according to the previous description [20, 21]. The images were observed using a BX51 light microscope (Olympus Corporation, Tokyo, Japan). Immunohistochemistry image analysis was performed using IPP6.0 software.

### 2.6. Western Blotting

The VE-cadherin and pY685 VE-cadherin were detected using a standard Western blot protocol to determine the VE-cadherin level and the phosphorylation of Y685. The transferred membrane was blocked with 10% skimmed milk for 1 h at room temperature, and then the blocked membrane was incubated with the primary antibody against VE-cadherin (dilution 1 : 1000; category number PA1-84328, Thermo Fisher), pY685VE-cadherin (dilution 1 : 700; category number AB119785, Abcam), and *β*-actin (dilution 1 : 700; Santa Cruz Biotechnology) overnight at 4°C, respectively. After incubation with the horseradish peroxidase-conjugated secondary antibody (dilutions of 1 : 5000; Beijing Zhongshan-Golden Bridge Biological Technology Company, Beijing, China) for 1 h at room temperature, the immunoblotting signals were visualized using a Western Luminescent Detection Kit (Vigorous Biotechnology, Beijing, China).

### 2.7. Immunofluorometric Assay

Immunofluorometric assay was carried out to determine the cellular skeleton. The coverslip wrapped by the PMVECs was fixed using paraformaldehyde, and then Actin-Tracker Green (1 : 100, BeoTime, C10330) was added followed by incubating at room temperature for 30 min in the dark. After washing with PBS containing 0.1% Triton X-100, the cells were counterstained. The mixture was incubated at room temperature for 30 min. Fluoromount-G (Southern Biotech, category number 0100-01, Birmingham, USA) was used to block the coverslip [22]. Finally, the images were observed using a BX53 fluorescence microscope (Olympus, Tokyo, Japan).

### 2.8. Permeability Assay

Transendothelial electrical resistance, an index of EC barrier function, was measured in real time using an electric cell substrate impedance sensing (ECIS) system (ECIS 1600, Applied BioPhysics) [23]. Cultured cells were plated on sterile eight-chambered gold-plated electrode arrays (8W10E plus) precoated with fibronectin and grown to full confluence. The electrode arrays were mounted on the ECIS system within an incubator (37°C, 5% CO_2_, 95% room air) and connected to its recorder device. Monolayer resistance was recorded at 15 kHz for 3 h in 5 min intervals.

### 2.9. Determination of Pulmonary Microvascular Permeability

The pulmonary microvascular permeability was determined through administration of azovan coerulen via caudal vein injection. The content of azovan coerulen was determined under a wave length of 625 nm, which was used to determine the extent of pulmonary microvascular permeability.

### 2.10. Determination of Wet-to-Dry Weight Ratios in Pulmonary Tissues

After sacrifice, lung tissues were obtained from the mice promptly in order to weigh the wet weight. Subsequently, the tissues were toasted at 70°C for 72 h until a constant weight. Afterwards, the wet-to-dry weight ratios were calculated. All the tests were carried out at least in triplicate.

### 2.11. Statistical Analysis

All statistical analysis was performed with SPSS 20.0. Quantitative data was presented as the mean ± SEM, at least from three independent experiments. Statistically significant differences in mean values were tested by Student's *t*-test or by one-way ANOVA using Dunnett's test in multiple comparisons. The difference of *P* value < 0.05 was considered statistically significant.

## 3. Results

### 3.1. Elevation of AngII in Blood Samples from AAD Complicated with ALI Patients

**Figure 1:**
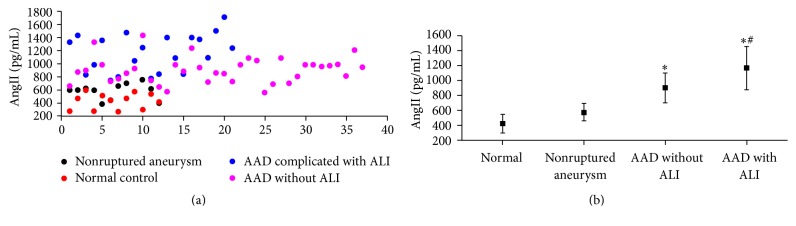
Circulating AngII is elevated in the blood samples from AAD complicated with lung injury patients. (a) AngII was assayed by the systems for each marker in the human peripheral blood samples from healthy control volunteers (*n* = 12); patients with nonruptured, chronic aortic aneurysm (*n* = 12); AAD patients without ALI (*n* = 37); or AAD complicated with ALI patients (*n* = 21). (b) The concentration of serum AngII in AAD complicated with lung injury patients was higher than the other groups. ^*∗*^*P* < 0.05 versus control, ^#^*P* < 0.05 versus AAD without ALI. AngII: Angiotensin II; AAD: acute aortic dissection.

The concentration of AngII in the patients with AAD complicated with ALI was remarkably elevated compared to the normal individuals and the AAD patients without ALI (Figure 1), which implied that AngII may be associated with the onset of AAD complicated with ALI.

### 3.2. AngII Contributed to the Elevation of Pulmonary Microvascular Permeability through Inducing Lung Injuries

**Figure 2:**
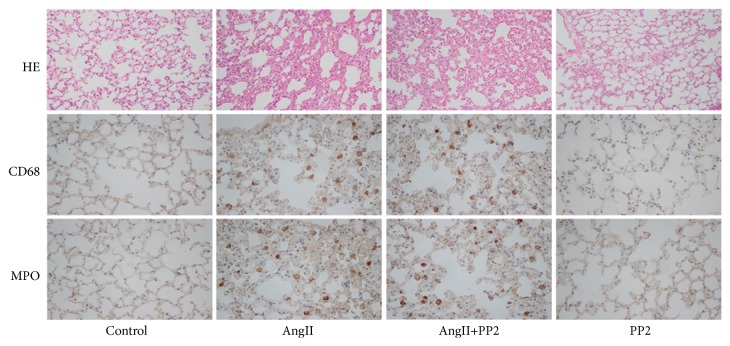
After establishing of acute lung injury model though minipumping of AngII, obvious edema was noticed in the pulmonary tissues in the AngII group, together with infiltration of neutrophils (MPO) and macrophages (CD68). However, after administration of PP2, no obvious changes were noticed in the infiltration of neutrophils and macrophages, as well as pulmonary edema compared with the AngII group. HE staining and immunohistochemistry images were observed under a magnification of 200x and 400x, respectively.

Based on the remarkable elevation of AngII in AAD complicated with ALI patients, in this study, we tried to induce ALI through minipumping of AngII. Besides, PP2 (Src kinase inhibitor) was given via intraperitoneal injection to determine the roles of Y685-VE-cadherin phosphorylation in the pulmonary microvascular barrier function and ALI mediated by AngII. HE staining and immunohistostaining showed obvious pulmonary intestinal edema in the mice model, together with massive infiltration of inflammatory cells in the lung tissues in the AngII group (Figure 2). Further, the pulmonary microvascular permeability and wet-to-dry (W/D) ratios in lung were remarkably elevated (Table 2). Nevertheless, in the AngII+PP2 group, no obvious changes were noticed in the infiltration of macrophages and neutrophils in the pulmonary tissues compared to the AngII group. Besides, no obvious changes were noticed in the pulmonary microvascular permeability and the W/D ratios between AngII+PP2 group and AngII group.

### 3.3. AngII Induced Increase of PMVECs Permeability

**Figure 3:**
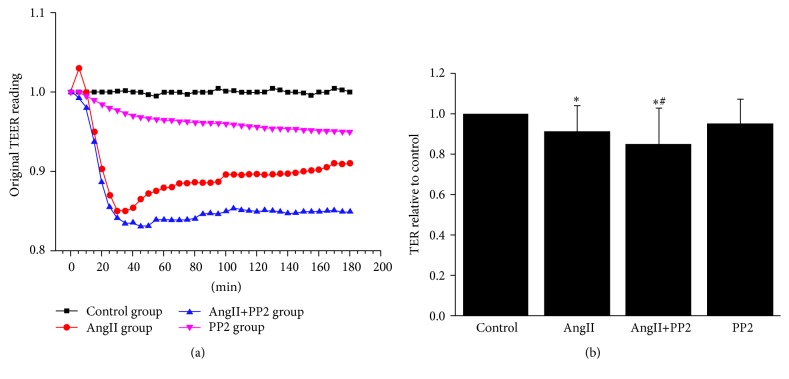
PMVECs were grown to confluence and quiescent monolayer, and transendothelial electrical resistance (TEER) was recorded. (a) The original TER recordings. (b) The relative TER in each group 3 h after interference (*n* = 3 per group). ^*∗*^*P* < 0.05 versus control group; ^#^*P* < 0.05 versus AngII group.

The pulmonary microvascular endothelial permeability was crucial for the pathogenesis of lung injury [24]. To investigate the AngII for the endothelial barrier function, the cultured PMVECs of four groups (i.e., control group, AngII group, AngII+PP2 group, and PP2 group) were subject to determination of monolayer PMVECs permeability using the electrical cell substrate impedance sensing (ECIS) system. In the confluent and quiescent monolayer, the transendothelial impedance in PMVECs was apt to decrease 10 minutes after AngII stimulation, which reached the minimal concentration at 30–40 min after stimulation. After administration of PP2, the transendothelial impedance showed continuous decrease (Figure 3), which indicated that AngII stimulation could contribute to the transient elevation of PMVEC permeability. However, such progression was increased continuously after inhibiting the Src kinase.

### 3.4. PMVEC Apoptosis Induced by AngII

**Figure 4:**
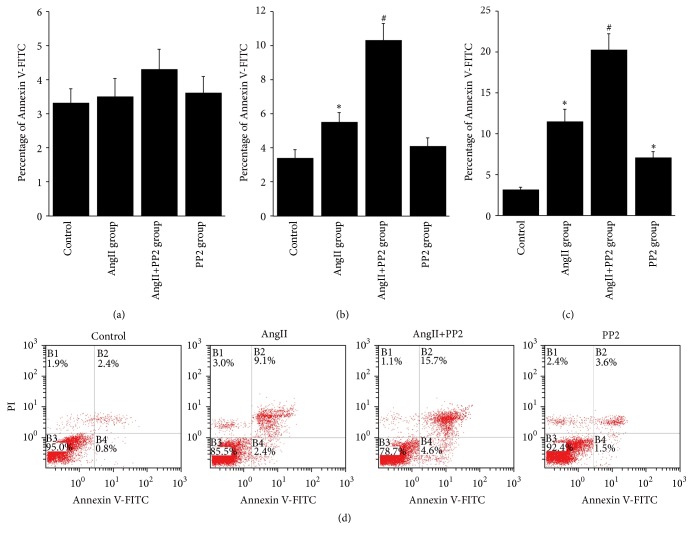
Determination of PMVECs apoptosis by flow cytometry. No obvious apoptosis was noticed in the PMVECs within 3 hours (a) after AngII stimulation. Apoptotic changes were noticed in the PMVECs within 12 h (b) to 24 h (c), which was obviously elevated after PP2 stimulation. (d) Obvious apoptotic changes were noticed in PMVECs within 24 h after AngII stimulation. ^*∗*^*P* < 0.05 versus control group; ^#^*P* < 0.05 versus AngII group.

Endothelial cell barrier injury was reported to be closely related to the endothelial apoptosis [25]. In this section, flow cytometry was carried out to determine the apoptosis of the cultured PMVECs in four groups (i.e., control group, AngII group, AngII+PP2 group, and PP2 group). No obvious apoptosis was induced within 3 h after AngII stimulation; however, remarkable apoptosis was noticed 12 h–24 h after stimulation, especially in the groups subject to PP2 interference. Interestingly, cellular apoptosis was noticed in the PP2 group at 12 h and 24 h, respectively (Figure 4). This indicated that cellular shrinkage and increase of intercellular space caused by endothelial skeletal rearrangement rather than endothelial apoptosis were the causing factor for the short-term increase of endothelial barrier permeability after AngII stimulation whereas, at the late stage after AngII stimulation, endothelial barrier injury and elevation of permeability were mainly associated with the obvious endothelial apoptosis. Taken together, PP2 may inhibit the Src kinase activity to increase the PMVEC apoptosis, but such phenomenon was not observed within 3 h after PP2 interference.

### 3.5. AngII Decreased the Level of pY685-VE-Cadherin and VE-Cadherin in Mice Lung Tissues

**Figure 5:**
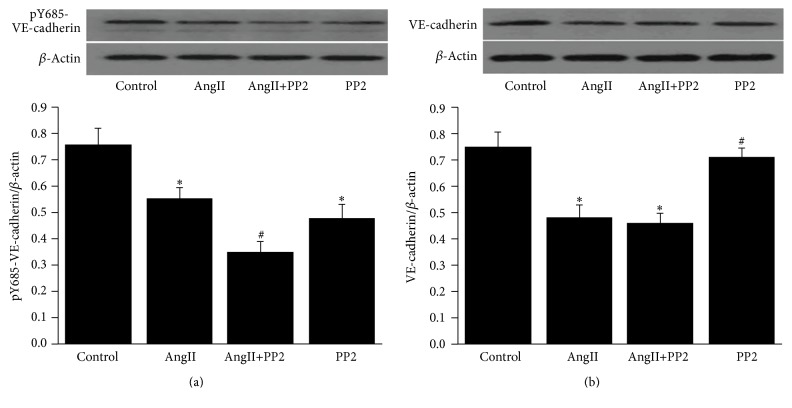
Downregulation of total pY685- VE-cadherin protein in lung induced by AngII. (a) The level of pY685-VE-cadherin was remarkably decreased in the AngII group compared with the control group. AngII+PP2 induced further downregulation of pY685-VE-cadherin, while the pY685-VE-cadherin was obviously reduced in the control group after PP2 interference. (b) The VE-cadherin was remarkably reduced in the AngII group compared with the control group, but the level of VE-cadherin showed no difference after application of pp2. ^*∗*^*P* < 0.05 versus control group; ^#^*P* < 0.05 versus AngII group.

To investigate the effects of AngII on the phosphorylation of pY685-VE-cadherin, Western blot analysis was performed. The results indicated that the Y685 of the VE-cadherin in the lung tissues of the control group was phosphorylated to some extent. However, after AngII stimulation, the level of pY685-VE-cadherin was remarkably decreased, especially in the AngII+PP2 group. Meanwhile, the pY685-VE-cadherin in the control group was also decreased after PP2 treatment (Figure 5(a)). Moreover, the level of VE-cadherin was also decreased significantly after AngII stimulation, while no obvious changes were noticed after PP2 treatment (Figure 5(b)). All these indicated that AngII may contribute to the dephosphorylation of Y685-VE-cadherin, but the downregulation of Y685-VE-cadherin may be related to the downregulation of VE-cadherin induced by AngII.

### 3.6. AngII Induced Dephosphorylation of Y685-VE-Cadherin in PMVECs

**Figure 6:**
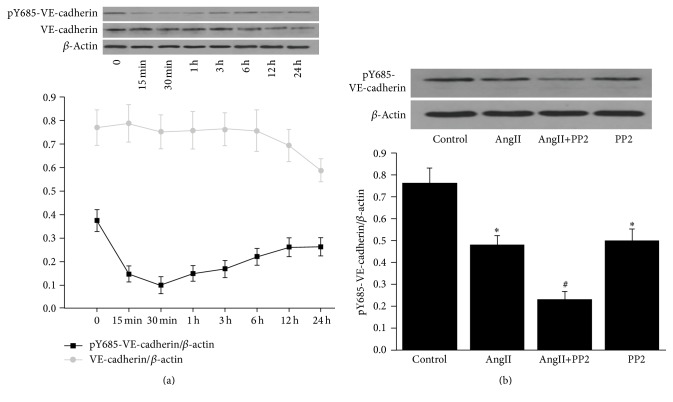
Expression of total pY685-VE-cadherin protein and VE-cadherin in cultured PMVECs. (a) The phosphorylation of Y685 of VE-cadherin induced by AngII in PMVECs was in a time-dependent manner. About 30 minutes after AngII stimulation, the dephosphorylation of Y685-VE-cadherin reached the peak level, whereas, 12 h after AngII stimulation, the VE-cadherin was decreased in PMVECs. (b) AngII or PP2 resulted in reduction of pY685-VE-cadherin in PMVECs, while the pY685-VE-cadherin was further decreased in the AngII+PP2 group. ^*∗*^*P* < 0.05 versus control group; ^#^*P* < 0.05 versus AngII group.

To determine the dephosphorylation of Y685-VE-cadherin induced by AngII, we determined the phosphorylation of Y685-VE-cadherin in PMVECs after AngII treatment. The results indicated that the Y685 of VE-cadherin was phosphorylated in PMVECs under physiological conditions. The dephosphorylation of Y685-VE-cadherin in PMVECs induced by AngII was in a time-dependent manner. To be exact, the level of pY685-VE-cadherin reached the lowest level about 30 minutes after AngII stimulation, but VE-cadherin was decreased about 12 h after AngII stimulation (Figure 6(a)). This indicated that AngII contributed to the dephosphorylation of Y685-VE-cadherin at the early stage. The pY685-VE-cadherin was obviously decreased about 1 h after AngII or PP2 stimulation. In AngII+PP2 group, the pY685-VE-cadherin was further reduced (Figure 6(b)). This indicated that the potential mechanisms in dephosphorylation of Y685-VE-cadherin induced by AngII and PP2 were different. Besides, the Src kinase was crucial for the phosphorylation of Y685-VE-cadherin.

### 3.7. Roles of Y685-VE-Cadherin Dephosphorylation in PMVEC Skeleton Rearrangement Induced by AngII

**Figure 7:**
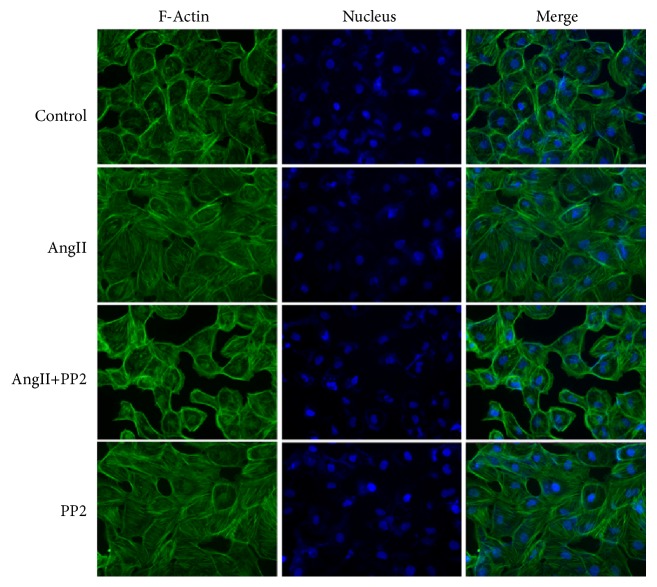
AngII induced skeletal rearrangement in PMVECs through inducing the dephosphorylation of Y685-VE-cadherin. Immunofluorescence indicated accumulation of stress fiber in PMVECs after AngII stimulation. In AngII+PP2 group, filopodia were noticed in the PMVECs, together with endothelial shrinkage and increase of intercellular space. The images were observed under a magnification of 400x.

Actin cytoskeletal rearrangements and perturbations have been implicated in other model systems as a critical, cell death independent mechanism of PMVEC barrier disruption [26, 27]. In addition, the phosphorylation of Y685 in VE-cadherin was closely related to the endothelial barrier dysfunction [28, 29]. Thus, we examined the morphology of PMVECs and the intracellular distribution and arrangement of F-actin fiber in fixed cells by phalloidin staining and fluorescence confocal microscopy.  Figure 7 shows that the F-actin fiber was mainly localized in the peripheral cell membrane in rat PMVECs. After AngII or PP2 stimulation, the pY685-VE-cadherin was decreased, and, at the same time, obvious changes were noticed in the morphology and distribution of F-actin fiber, together with presence of stress fiber in the cells. In AngII+PP2 group, the pY685-VE-cadherin was further decreased. Filopodia were noticed in the PMVECs, together with endothelial shrinkage and increase of intercellular space. Taken together, we speculated that dephosphorylation of Y685-VE-cadherin may be closely related to the accumulation and rearrangement of cellular skeleton.

## 4. Discussion

The mechanism of AAD complicated with ALI is still not well defined. Several factors have been reported to be related to such disease, including systemic acute inflammatory reaction, serum MMP-9, body temperature, and the scale of AAD [2, 4, 30, 31]. Our results indicated that AngII level was higher in the AAD patients complicated with ALI compared with those without ALI or normal individuals. On this basis, we speculate that AngII may involve in the pathogenesis of AAD complicated with ALI. We established a mice ALI model through pumping of AngII, in which the pulmonary microvascular permeability was increased together with massive infiltration of inflammatory cells and obvious edema. Taken together, we speculated that AngII may induce ALI through impairing the pulmonary microvascular endothelial barrier.

VE-cadherin, consisted of 780 amino acids, is an important component for the vascular endothelial adhesion [32, 33]. In structure, the VE-cadherin consisted of extracellular region, intracellular region, and the transmembrane region. The extracellular region could adhere to the adjacent VE-cadherin in the vascular endothelial cells, while the intracellular region could adhere to the P120-catenin, *β*-catenin, and plakoglobin. Besides, it could form the VE-cadherin complex together with the *α*-catenin and F-actin [34–36], which played important roles in the endothelial barrier function as through the condensed adhesion of the vascular endothelial cells [10, 36].

The phosphorylation of tyrosine in the intracellular region of the VE-cadherin was crucial for the stability of VE-cadherin complex. Stimulation of ICAM-1 leads to phosphorylation of VE-cadherin, which is a prerequisite for adherens junction disassembly. In HUVECs, the kinases Src and Pyk2 phosphorylate VE-cadherin on the p120 and *β*-catenin binding sites tyrosine residues 658 and 731, respectively. This inhibits the binding of p120 and *β*-catenin to VE-cadherin 33. Because the interaction of these proteins with VE-cadherin is critical for retaining VE-cadherin at the adherens junction, this destabilizes the junctions. Besides, Rac1 activation leads to phosphorylation of VE-cadherin on serine 665, which signals its clathrin-dependent internalization [37].

Recent studies revealed that the phosphorylation of Y685-VE-cadherin was related to the dynamic state of adherens junctions and vascular endothelial permeability under the stimulation of inflammatory factors [28, 29]. Wallez et al. revealed that VEGF-induced-VE-cadherin tyrosine phosphorylation was mediated by Src on Y685, a process that appeared to be critical for VEGF-induced endothelial cell migration [28]. Sidibé et al. reported that vascular endothelial growth factor induced VE-cadherin tyrosine phosphorylation at Y685 in a Src-dependent manner and the site Y685 in VE-cadherin was involved in the physiological regulation of capillary permeability [38]. Moreover, Orsenigo et al. revealed that VE-cadherin was phosphorylated in Y658 and Y685 in vivo in veins under resting conditions. Furthermore, bradykinin could induce phosphorylation of Y658 and Y685 of VE-cadherin, which resulted in internalization and ubiquitination of phosphorylated VE-cadherin and elevation of vascular permeability, whereas in vitro study showed that point mutation of Y658F and Y685F contributed to the prevention of vascular endothelial cadherin internalization, ubiquitination, and elevation in permeability by bradykinin [29].

Our study revealed VE-cadherin was phosphorylated in Y685 in PMVECs under resting conditions. The pY685-VE-cadherin was decreased in PMVECs 15 min after AngII stimulation and reached the minimal level about 30 min after stimulation. However, the level of VE-cadherin began to decrease about 12 h after AngII stimulation. These indicated that AngII could induce dephosphorylation of Y685-VE-cadherin in PMVECs. After PP2 (Src kinase inhibitor) treatment, the level of pY685-VE-cadherin was further inhibited, which indicated that Src kinase might involve in the phosphorylation of Y685-VE-cadherin.

Endothelial barrier function was mainly related to the endothelial cell apoptosis and cellular skeleton rearrangement [25–27]. In this study, no obvious cellular apoptosis was observed in PMVECs 3 h after AngII stimulation, but aberrant changes were noticed in the cellular skeleton together with cellular shrinkage. This may explain the increased endothelial permeability of AngII at the early stage, whereas, in the PMVECs stimulated by AngII, apoptotic changes were noticed about 12 h after stimulation, which might be responsible for the increased endothelial permeability after AngII stimulation at late stage.

The dephosphorylation of Y685-VE-cadherin was shown to be related to the skeletal rearrangement in the PMVECs. AngII may induce dephosphorylation of Y685-VE-cadherin in PMVECs, together with obvious changes in the morphology and distribution of F-actin, as well as accumulation of stress fiber, whereas, after PP2 treatment, the dephosphorylation of Y685-VE-cadherin was further increased, and aberrant changes were noticed in the endothelial cell morphology and elevation of skeletal rearrangement in PMVECs, as well as increase of cellular permeability.

In conclusion, the effect of AngII on dephosphorylation Y685-VE-cadherin is an important mechanism of regulation of VE-cadherin biological functions. A salient finding of the present paper is that AngII could increase the endothelial barrier permeability through inducing endothelial skeletal rearrangement at early stage, in which the dephosphorylation Y685-VE-cadherin induced by AngII played a crucial role in such biological process. These results suggest that dephosphorylation of Y685 in VE-cadherin in response to AngII may be a key player in the endothelial barrier dysfunction induced by AngII. These findings provide new strategies for the management of AAD complicated with ALI. Besides, these findings approved that Src kinase was crucial for the phosphorylation of Y685-VE-cadherin with Y685 serving as a target of Src. However, we cannot exclude the possibility that inhibiting Src kinase may trigger other endogenous protective mechanisms as PP2 causes no effects on the deterioration of lung injury caused by AngII in mice.

## Figures and Tables

**Table 1:** Clinical features of the patients.

	Control (*n* = 12)	Nonruptured aneurysm (*n* = 12)	AAD	
			Complicated with lung injury AAD (*n* = 21)	Nonlung injury AAD (*n* = 37)
Age, yr	40 ± 9	58 ± 11	47 ± 6	51 ± 6
Male sex, *n*%	8 (66.7)	8 (66.7)	17 (81.0)	30 (81.1)
Average duration from onset, h	N/A	N/A	9.2	10.7
Smoking, *n* (%)	4 (33.3)	5 (41.7)	11 (52.4)	19 (51.4)

**Table 2:** Determination of pulmonary microvascular permeability and W/D in each group.

Group	Pulmonary microvascular permeability (*μ*g/mg)	W/D
Normal control	0.047 ± 0.003	3.19 ± 0.48
AngII group	0.106 ± 0.08^*∗*^	4.72 ± 0.48^*∗*^
AngII+PP2 group	0.117 ± 0.06^*∗*^	4.63 ± 0.55^*∗*^
PP2 group	0.048±0.004	3.37 ± 0.29

^*∗*^ *P* < 0.05, compared with normal control.
